# Spike-timing relationship of neurochemically-identified dorsal raphe neurons during cortical slow oscillations

**DOI:** 10.1016/j.neuroscience.2011.08.072

**Published:** 2011-11-24

**Authors:** J.V. Schweimer, N. Mallet, T. Sharp, M.A. Ungless

**Affiliations:** a Medical Research Council Clinical Sciences Centre, Imperial College London, Hammersmith Hospital, Du Cane Road, London W12 0NN, UK; b Department of Pharmacology, University of Oxford, Mansfield Road, Oxford, OX1 3QT, UK; c Medical Research Council Anatomical Neuropharmacology Unit, University of Oxford, Mansfield Road, Oxford, OX1 3TH, UK

**Keywords:** 5-HT, serotonergic, dopamine, basal ganglia, limbic system, ANOVA, analyses of variance, COV-IS, coefficient of variation of the inter-spike-interval, DRN, dorsal raphe nucleus, ECoG, electrocorticogram, PBS, phosphate-buffered saline, PBS-X, PBS containing 0.2% Triton X-100, PFC, prefrontal cortex, SWA, slow-wave activity, TH, tyrosine hydroxylase, 5-HT, 5-hydroxytryptamine

## Abstract

The firing activity of dorsal raphe neurons is related to arousal state. However, it is unclear how this firing activity is precisely related to cortical activity, in particular oscillations occurring during sleep rhythms. Here we conducted single-cell extracellular recordings and juxtacellular labelling while monitoring electrocorticogram (ECoG) activity in urethane anaesthetised rats, to relate activity in neurochemically identified groups of neurons to cortical slow-wave activity (SWA). We observed that electrophysiological heterogeneity in dorsal raphe neurons revealed different neurochemical groups of DRN neurons and was mirrored by significant differences in the phase and strength of coupling to the cortical slow oscillations. Spike firing relationship of clock-like neurons, identified as 5-HT (5-hydroxytryptamine) or serotonin neurons, was higher during the inactive component of the oscillations. In contrast, half of the identified bursting 5-HT neurons did not exhibit strong cortical entrainment; those that did fired most during the inactive component of the SWA. Two groups of putatively non-5-HT neurons (irregular slow-firing and fast-firing) exhibited significant coherence and fired most during the active component of the SWA. These findings indicate that within the DRN electrophysiologically and neurochemically discrete neuronal groups exhibit distinct relations to cortical activity.

## Highlights

▶DRN neurons exhibit heterogeneous firing in relation to cortical oscillations. ▶Clock-like 5-HT neurons fire most during inactive component of the oscillation. ▶Half of bursting 5-HT neurons did not exhibit coupling to the oscillation. ▶Non-5-HT neurons fired most during the active component of the oscillation.

Serotonin or 5-HT (5-hydroxytryptamine) in the midbrain dorsal raphe nucleus (DRN) has been implicated in many aspects of behavioural and cognitive function, including movement, punishment, cognition, and in particular regulation of the sleep–wake cycle (Bradley, 1958; Jouvet, 1972; Jacobs and Fornal, 1999; Meneses, 1999; Sakai and Crochet, 2001; Steriade, 2004; Dayan and Huys, 2009; Monti, 2010). Putative DRN 5-HT neurons, across species, fire regularly at a relatively high rate during wakefulness but decrease in firing during slow-wave sleep and become silent during paradoxical or rapid-eye movement sleep (Trulson and Jacobs, 1979; Shima et al., 1986; Jacobs and Azmitia, 1992; Kocsis and Vertes, 1992; Portas et al., 2000; Urbain et al., 2006). In a similar fashion, regional brain extracellular 5-HT measured by microdialysis decreases during the switch from wakefulness to slow-wave and paradoxical sleep (Wright et al., 1990; Portas et al., 1998).

During slow-wave sleep there are large-scale changes in neural activity in the cortex, in which the membrane potential of many groups of cortical neurons fluctuate between depolarised (up) and hyperpolarised (down) states in a highly synchronised manner to generate large amplitude, low frequency slow waves (Steriade, 2000). This slow-wave activity (SWA) is present during natural sleep and can also be induced by certain anaesthetics like urethane. SWA is expressed as cortical slow oscillation (∼1 Hz), as well as ∂ (1–4 Hz) and spindle (7–14 Hz) oscillations (Steriade, 2000).

Neuronal pathway tracing studies demonstrate that the cortex, and especially the prefrontal cortex (PFC), has strong reciprocal neuroanatomical connections with the DRN (Hajós et al., 1998; Heidbreder and Groenewegen, 2003; Gonçalves et al., 2009). Moreover, *in vivo* electrophysiological studies demonstrate that these projections are powerful, with significant numbers of DRN neurons responding to PFC stimulation (Hajós et al., 1998; Varga et al., 2001) and significant numbers of PFC neurons responding to DRN stimulation (Gartside et al., 2000). Recent behavioural, pharmacological, and also electrophysiological evidence suggest a top–down control of the PFC over 5-HT and other monoamine systems via its descending projections (see Robbins, 2005), and thus an association between cortical SWA and firing of DRN 5-HT neurons is predicted.

In support of this idea, there is considerable diversity in the firing activity of individual DRN neurons across different sleep–wake states (Kocsis and Vertes, 1992; Sakai and Crochet, 2001; Kocsis et al., 2006; Urbain et al., 2006). However, interpretation of this diversity is complicated by not only the variety of firing patterns expressed by DRN neurons but also the many chemically distinct DRN neuron types. For example, recent findings using juxtacellular labelling methodology have found that many but not all classic slow-firing clock-like neurons are 5-HT-containing (Allers and Sharp, 2003; Schweimer and Ungless, 2010). Furthermore, not all chemically identified 5-HT neurons in the DRN are regular and slow-firing; there is a subpopulation of bursting 5-HT neurons (Hajós et al., 2007; Schweimer and Ungless, 2010) as well as subpopulations of fast-firing 5-HT neurons (Allers and Sharp, 2003; Kocsis et al., 2006).

The present study investigated the relationship between DRN neuron firing and cortical activity by combining single-cell recording and juxtacellular labelling in the DRN whilst monitoring electrocorticogram (ECoG) activity in anaesthetised rats. This has allowed for the first time, electrical activity of distinct chemically identified DRN neurons to be correlated with measurements of cortical SWA.

## Experimental procedures

### Animals

A total of 35 male Sprague–Dawley rats (250–450 g, Charles River, Margate, Kent, UK) were used. They were housed collectively with *ad libitum* access to food and water and maintained on a 12-h light/dark cycle. All experiments were conducted in accordance with the Animals (Scientific Procedures) Act 1986 (UK) and associated guidelines.

### Electrophysiological recordings and juxtacellular labelling

General anaesthesia was induced with isoflurane (Isoflu, Abbott, Queenborough, Kent, UK) and maintained with urethane (1.3–1.5 mg/kg, ethyl carbamate, Sigma, Steinheim, Germany), plus supplemental doses of ketamine (30 mg/kg, i.m.; Ketaset, Willow Francis, Crawley, West Sussex, UK) and xylazine (10 mg/kg, i.m.; Rompun, Bayer, Newbury, Berkshire, UK) as required. Animals were placed in a stereotaxic frame (David Kopf Instruments, Tujunga, CA, USA), and the skull was prepared for intracerebral recordings. All wound margins were infiltrated with the local anaesthetic lidocaine, and corneal dehydration was prevented by applications of Hypromellose eye drops. Body temperature was maintained at 37±0.5 °C using a homeothermic heating blanket (Harvard Apparatus, Kent, UK).

The ECoG was recorded via a steel screw over the left frontal cortex (2.7 mm anterior, 2.0 mm lateral in relation to bregma; Paxinos and Watson, 2007) and referenced against a second steel screw positioned over the ipsilateral cerebellum. Raw ECoG was band-pass filtered (0.3–1500 Hz, −3 dB limits) and amplified (2000×, NL104 preamplifier, Digitimer, Welwyn Garden City, UK) before acquisition. Craniotomy was performed above the DRN (7–8 mm posterior to bregma, over the midline according to Paxinos and Watson, 2007); special care was taken to avoid damage to the underlying midsagittal sinus. Mineral oil was applied to the exposed brain surface to prevent dehydration.

Extracellular neuronal activity was monitored with a 15–25 MΩ glass microelectrode filled with 1.5% Neurobiotin (Vectorlabs, Burlingame, CA, USA) in 0.5 M NaCl (tip diameter 1–1.5 μm). Electrode signals were alternating current (AC)-coupled, amplified (1000×), and band-pass filtered (0.3–5 kHz) using a Neurolog system (Digitimer, Welwyn Garden City, Hertfordshire, UK) and acquired on-line through a Micro1401 interface and Spike2 software (Cambridge Electronic Design, Cambridge, Cambridgeshire, UK). Mains noise at 50 Hz was eliminated (‘Humbug’ filter; Brown et al., 2002) for single unit and ECoG recordings. Neurons were subsequently filled with neurobiotin using the juxtacellular labelling method (Pinault, 1996; Allers and Sharp, 2003; Schweimer and Ungless, 2010). Briefly, positive current pulses were applied through the microelectrode (200 ms; 2.5 Hz; 1–5 nA). The amount of current applied was continuously monitored and adjusted to obtain modulation of neuronal activity as this enabled detectable labelling of the soma and dendrites of the neuron with neurobiotin.

### Immunohistochemistry

Following juxtacellular labelling, animals were transcardially perfused with phosphate-buffered saline (PBS) followed by 4% paraformaldehyde. Brains were removed and kept in 4% paraformaldehyde overnight, before being transferred to a 30% sucrose solution for cryoprotection. Coronal sections (20 μm) were cut using a cryostat (CM 1800, Leica Microsystems, Wetzlar, Germany). Sections were then stained using a standard protocol for free-floating sections. Following several rinses in PBS containing 0.2% Triton X-100 (PBS-X), sections were incubated in blocking solution (PBS-X with 6% normal donkey serum) for at least 30 min, and then incubated overnight at room temperature in primary antibody solution (PBS-X containing 2% normal donkey serum and rabbit anti-5-HT (1:2000, Immunostar 20800, Hudson, WI, USA), and mouse anti-tyrosine hydroxylase (TH; 1:1000, Sigma T-2928, Gillingham, Dorset, UK)). Sections were subsequently washed and incubated in secondary antibody solution [PBS-X containing 2% normal donkey serum, CY3-conjugated streptavidin (1:1000, Jackson ImmunoResearch, Newmarket, Suffolk, UK), Alexa 488-conjugated donkey anti-rabbit (1:2000, Invitrogen Ltd., Paisley, Renfrewshire, UK), Alexa 350- or 405-conjugated goat anti-mouse (1:250 or 1:1000, Invitrogen Ltd.)] for 90 min at room temperature. Sections were then rinsed in PBS and mounted on slides for examination under a confocal microscope (SP1, Leica Microsystems, Germany) using Leica LCS software.

Antibody specificity was evident in that 5-HT and TH antibody labelling was restricted to regions previously shown to contain 5-HT and dopaminergic neurons. Moreover, no double labelling for 5-HT and TH was observed and no immunolabelling was observed in control experiments in which either primary or secondary antibodies were omitted.

Images were cropped to illustrate the region of interest, and brightness and contrast adjusted using Leica LCS or ImageJ64 software (Rasband, W.S., ImageJ, USA. National Institutes of Health, Bethesda, MD, USA, http://imagej.nih.gov/ij/, 1997–2011). DRN subdivisions and stereotaxic coordinates are based on those found in Paxinos and Watson (2007).

### Data analysis

The firing rate frequency and the coefficient of variation of the inter-spike-interval (COV-IS; measure of regularity) were calculated for each neuron. Action potential waveform duration was measured from the onset of the action potential (defined as a change of more than 0.02 mV from baseline) to the negative trough and the biphasic waveform (i.e. return to zero). Single-unit activity was recorded during cortical SWA, which accompanies deep anaesthesia and resembles activity observed during natural sleep. A typical baseline period of 3 min with SWA and co-registered single-unit activity was recorded for each neuron. After physiological characterization, several recorded neurons were juxtacellularly labelled per animal and subsequently processed for immunocytochemistry.

ECoG data were visually inspected and only epochs of robust cortical SWA were selected for analysis (Magill et al., 2000). The “active component” of the slow wave oscillation is expressed by a highly synchronous spike discharges in cortical neurons resulting in a long peak with spindle activity. The trough in the slow oscillations during which high-frequency oscillations are weakest, or absent, is referred to as the ‘inactive component’. This terminology is used in preference to ‘up’ and ‘down’ states, as these generally refer to membrane states of individual neurons as recorded intracellularly (Mallet et al., 2008; Mena-Segovia et al., 2008). Autocorrelations of single-unit activity were calculated. Coherence between the ECoG and single-unit activity was used to assess correlations in the frequency domain. The coherence was examined for the frequency domains of the slow oscillations (0.5–1.5 Hz), the spindle activity (7–14 Hz), and gamma oscillations (30–60 Hz). These calculations were performed using scripts within the Spike 2 software (CED, version 6, Cambridge, Cambridgeshire, UK).

Activity histograms were calculated to determine the phase relationship between the DRN single-unit activity and cortical slow wave oscillations (active vs. inactive components). To improve ‘peak’ (active component) and ‘trough’ (inactive component) detection, ECoG signals were filtered (∼1 Hz) to retain slow oscillations and exclude high frequencies. Using custom MATLAB routines, the peak and troughs of the cortical slow oscillation were detected automatically based on the time of zero crossing and taking into account the average time-width of peaks and troughs. Periods of weaker slow oscillations were filtered out by the MATLAB routine (for details see Mallet et al., 2008). After defining active or inactive components of ECoGs, coincident spikes were automatically assigned to one of 14 bins (7 bins each for active and inactive components; MATLAB). Spike counts per bin across all accepted oscillation components were then normalized (by converting to firing rate) to take into account the variable durations of active and inactive components, and then displayed in an activity histogram.

### Statistical analysis

Baseline firing parameters (firing rate, COV-IS, action potential width) of the DRN neuron subgroups (regular 5-HT, bursting 5-HT, irregular-, and fast-firing neurons) were compared using one-way analyses of variance (ANOVA). When the assumption of the homogeneity of variances was violated, a Brown–Welch *F*-test was used with a subsequent Games–Howell post hoc test. Mean peak coherence values of the main frequency domains (0.5 to 1.5 Hz) of the different neuron subgroups were compared using a one-way ANOVA followed by a least significant difference (LSD) post hoc test. Probability values of less than 5% were considered statistically significant. All statistical calculations were carried out using IBM SPSS Statistics for Mac Version 19 (Chicago, IL, USA). Mean±SEM values are shown throughout.

## Results

### Electrophysiological and neurochemical properties of DRN neurons

A total of 71 DRN neurons were recorded during robust cortical slow oscillations (baseline recording times >3 min), 21 of which were juxtacellularly labelled (Pinault, 1996) and found to be located within the ventral and dorsal parts of the DRN (between −6.9 mm to −8.2 mm from bregma). Of these labelled neurons, 14 were immunopositive for 5-HT only, one for TH only, and four neurons were immunonegative for both 5-HT and TH. One animal presented with multiple labelled neurons, and another had neurons with insufficient staining of 5-HT or TH (in both cases neurons were classified as unlabelled).

**Fig. 1:**
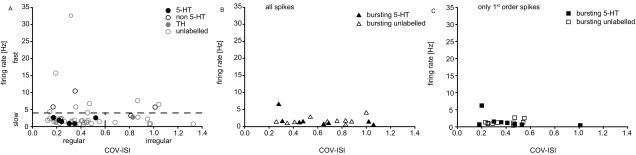
DRN neuron diversity. DRN neurons have been grouped according to their electrophysiological properties. (A) Neurons were divided into slow (≤4 Hz) and fast firing neurons. The slow neurons were further divided into regular or clock-like (COV-IS<0.6) and irregular neurons. In the slow, clock-like (regular) category, all neurochemically identified neurons were 5-HT (6/6, black filled circles). Slow, irregular firing neurons were of either TH (grey filled circle) or non-5-HT (black unfilled circle); fast firing neurons, when identified were also non-5-HT (black unfilled circles). (B) Bursting neurons were identified by stereotypical bursts comprising with spike doublets or triplets with the second or third-order spikes typically occurring within <10 ms. Generally they were slow-firing, with diverse regularity, all neurochemically identified neurons in this group (*n*=8, black filled triangles) were immunopositive for 5-HT but not TH. (C) Firing rate and COV-IS of bursting neurons when only first-order spikes are analysed was similar to clock-like neurons.

On the basis of their waveform characteristics, firing frequency and regularity, four electrophysiologically distinct groups of DRN neurons were identified (see Fig. 1A, B); (i) ‘clock-like’ neurons with broad spike waveforms fired in a slow (≤4 Hz) and regular (COV-IS of <0.6) firing pattern (49.3% of total recorded neurons) (Fig. 1A), (ii) neurons with broad spike waveforms fired in a slow firing pattern but exhibiting a stereotypical bursting (25.4% of total), and therefore with a more irregular firing pattern (see Hajós et al., 2007; Schweimer and Ungless, 2010) (Fig. 1B and C), (iii) neurons with a high (>4 Hz) firing rate (14.1% of total) (Fig. 1A), and (iv) neurons with a slow but irregular firing pattern (COV-IS>0.6) without stereotypical burst-firing (11.3% of total) (Fig. 1A).

**Fig. 2:**
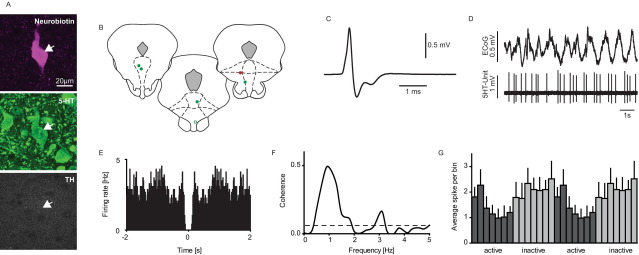
Electrophysiological and neurochemical properties of DRN clock-like neurons. A representative example of a clock-like 5-HT neuron (A), this neuron was juxtacellularly labelled and immunohistochemically identified as serotonergic (scale bar: 20 μm; 5-HT, serotonin; TH, tyrosine hydroxylase). (B) Coronal sections showing the localisation of all neurochemically identified clock-like 5-HT neurons within the DRN (the unfilled circle is the illustrated example neuron; adaptations from Paxinos and Watson (2007), Figs. 91, 93, and 96; −6.96 to −7.56 mm posterior to bregma). Green circles indicate labelled 5-HT clock-like neurons, which predominantly fired during the inactive component of the cortical slow oscillations. The red circle indicates a clock-like 5-HT neuron, which fired predominantly during the active component. (C) The representative example neuron exhibited a broad averaged extracellular waveform, (D) a slow regular firing pattern which is time-linked to the cortical slow oscillations. (E) Autocorrelation of the unit firing during SWA (bin size=10 ms). (F) Significant coherence values between cortical and DRN signals in the predominant frequencies domains of the cortical slow oscillations (0.5–1.5 Hz); (G) Activity histogram (see Experimental procedures) of all juxtacellularly labelled regular-firing 5-HT neurons (*n*=6), showing a higher firing activity during the inactive component of the cortical slow oscillations (mean±SEM). For interpretation of the references to color in this figure legend, the reader is referred to the Web version of this article.

The majority of neurons encountered (*n*=35/71) were slow firing ‘clock-like’ neurons, classically assumed to be 5-HT-containing (Figs. 1A and 2D). Attempts to juxtacellularly label 18 neurons in this group were successful for six neurons, all of which were found to be immunopositive for 5-HT, and immunonegative for TH (Fig. 2A, B).

**Fig. 3:**
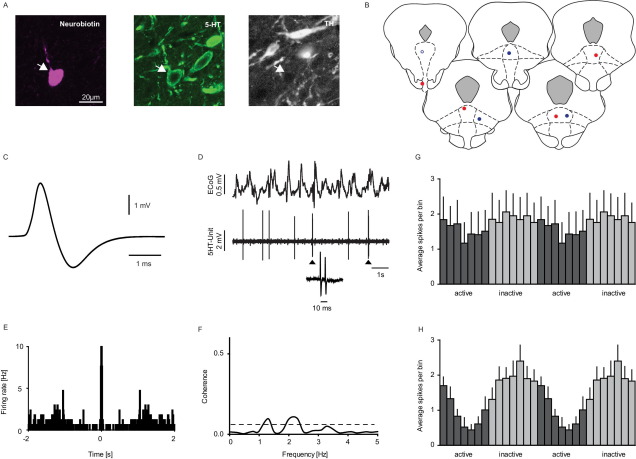
Electrophysiological and neurochemical properties of DRN bursting neurons. A representative example of a bursting 5-HT neuron (A). This neuron was juxtacellularly labelled and immunohistochemically identified as serotonergic (scale bar: 20 μm; 5-HT, serotonin; TH, tyrosine hydroxylase). (B) Coronal sections showing the localisation of all neurochemically identified bursting 5-HT neurons within the DRN (the unfilled circle is the illustrated example neuron; adaptations from Paxinos and Watson (2007), Figs. 91, 93, and 96–98; −6.96 to −7.80 mm posterior to bregma). Blue circles indicate labelled 5-HT bursting neurons, whose firing was not coherent to the cortical slow oscillations; the red circles indicate bursting 5-HT neurons whose firing was linked to the cortical slow oscillations. (C) The representative bursting 5-HT neuron had a broad averaged extracellular waveform and (D) fired independently of the SWA; the arrows mark spikes, which fired as doublets indicated by the inlay, (E) Autocorrelation of the unit firing during SWA (bin size=10 ms); the peak indicates the fast 5-HT bursts. (F) No significant coherence (minimum of two significant bins is necessary) between the cortical and DRN activity was detected in the 1 Hz frequency domain. (G) Activity histogram (see Experimental procedures) of all non-coherent bursting neurons (*n*=10), (H) activity histogram of all coherent bursting neurons (*n*=8), which fired predominantly during the inactive component of the cortical slow oscillations. For interpretation of the references to color in this figure legend, the reader is referred to the Web version of this article.

The second main neuron group (*n*=18/71) were not significantly different to clock-like neurons in terms of their slow firing rate and broad spike waveform (*P*>0.05; see Figs. 1B, C and 3) but were less regular (*P*<0.001; Fig. 1B and C). Most notably these neurons displayed stereotypical bursts comprising with spike doublets or triplets with the second or third-order spikes typically occurring within less than 10 ms (inter spike interval 7.98±0.48 ms) following the first-order spike (% of spikes in doublets 26.23±6.45%; Fig. 3D, E). When regularity of these neurons was calculated including only first-order spikes, their COV-IS was not different from ‘clock-like’ neurons (*P*>0.05; Fig. 1C). Attempts to juxtacellular label eight such neurons were all successful and all neurons were immunopositive for 5-HT, but immunonegative for TH (Fig. 3A, B). For the subsequent analysis data from labelled and unlabelled bursting neurons were pooled.

**Fig. 4:**
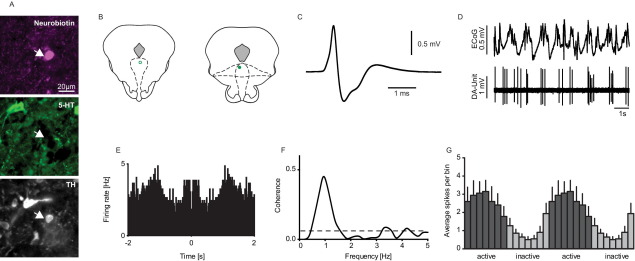
Electrophysiological properties of irregular non-serotonergic DRN neurons. A representative example of an irregular-firing DRN neuron (A), this neuron was juxtacellularly labelled and immunohistochemically identified as immunopositive for TH (scale bar: 20 μm; 5-HT, serotonin; TH, tyrosine hydroxylase). (B) Coronal sections showing the localization of all labelled irregular-firing neurons within the DRN (the unfilled circle is the illustrated example neuron; adaptations from Paxinos and Watson (2007), Figs. 91 and 93, −6.96 to −7.08 mm posterior to bregma). (C) Extracellular waveform average. (D) The firing of this neuron is time-linked to the cortical SWA. (E) Autocorrelation of the single unit activity (Bin size: 10 ms). (F) Significant coherence values between cortical and DRN signals in the predominant frequencies domains of the cortical slow oscillations (0.5–1.5 Hz). (G) The activity histogram (see Experimental procedures) of all recorded irregular, non-5-HT neurons (*n*=8). These neurons fired predominantly during the active phase of the SWA. For interpretation of the references to color in this figure legend, the reader is referred to the Web version of this article.

The third neuron group (*n*=8/71) were slow firing at a rate not significantly different from clock-like (*P*>0.05, Figs. 1A and 4) but with a less regular firing pattern (*P*<0.001, Figs. 1A and 4D) and a narrower spike waveform (*P*<0.001, Fig. 4C). Juxtacellular labelling was attempted in four neurons, and successful in two cases; one was immunopositive for TH but not 5-HT (Fig. 4A, B) while the other was immunonegative for both 5-HT and TH.

**Fig. 5:**
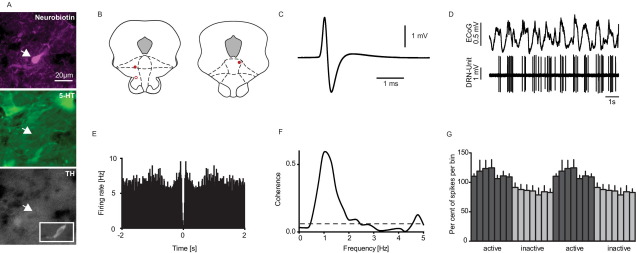
Electrophysiological properties of fast-firing non-serotonergic DRN neurons. A representative example of a fast-firing DRN neuron (A), this neuron was juxtacellularly labelled and immunohistochemically identified as non-5-HT/non-TH (scale bar: 20 μm; 5-HT, serotonin; TH, tyrosine hydroxylase). The insert shows a TH immunopositive cell in the same focal plane elsewhere in the section. (B) Coronal sections showing the localisation of all labelled fast-firing neurons within the DRN (the unfilled circle is the illustrated example neuron; adaptations from Paxinos and Watson (2007), Figs. 93 and 96, −7.08 to −7.44 mm posterior to bregma). (C) Narrow extracellular waveform average. (D) The firing of this neuron is time-linked to the cortical SWA. (E) Autocorrelation of the single unit activity (Bin size: 10 ms). (F) Significant coherence values between cortical and DRN signals in the predominant frequencies domains of the cortical slow oscillations (0.5–1.5 Hz). (G) The activity histogram (see Experimental procedures) of all fast-firing neurons, which showed significant coherence between the non-5-HT neuron (*n*=10), these fired predominantly during the active phase of the SWA. For interpretation of the references to color in this figure legend, the reader is referred to the Web version of this article.

The fourth neuron group (*n*=10/71) had a consistently high firing rate (*P*<0.05) and a narrower spike waveform compared to the clock-like neurons (*P*<0.01, Figs. 1A and 5C) but a large variability in regularity (COV-IS range 0.14–1.04, Fig. 1A). Juxtacellular labelling was attempted in five neurons, with three being successful, and all cases were immunonegative for both 5-HT and TH (Fig. 5A, B).

### Correlation of rhythmic cortical activity with firing of 5-HT DRN neurons

Under deep urethane anaesthesia SWA in the cortex was expressed in the ECoG traces by large amplitude (>400 μV) slow oscillations (∼1 Hz). The firing of most clock-like DRN neurons examined (*n*=33/35) demonstrated significant coherence (at least two bins over the calculated significance level of 0.065) with cortical slow oscillations in the 0.5–1.5 Hz frequency domains, which predominated the cortical slow oscillations. The vast majority of the clock-like neurons (*n*=28/35, 80%) had higher firing during the inactive component of the slow oscillations as indicated by the activity histograms (Fig. 2B–F). However, a small number of clock-like neurons (*n*=5/35, 14.3%) had higher firing during the active component of the slow oscillations. All six neurochemically identified 5-HT neurons with clock-like activity exhibited coherence with the slow oscillations, and five out of six had higher firing during the inactive component (Fig. 2G).

In comparison to the clock-like neurons, around half of the burst-firing DRN neurons (*n*=8/18, 44%) also exhibited significant coherence, with firing being highest during the inactive component of slow cortical oscillations (see Fig. 3H). However, half (*n*=10/18, 56%) of the bursting neurons did not exhibit significant coherence between their firing and cortical slow oscillations (see Fig. 3D–G). Of the identified bursting 5-HT neurons, half (four out of eight) showed coherence, which was linked to the inactive component of the slow oscillations. These two groups of burst-firing neurons did not differ in terms of firing rate (coherence (mean±SEM): 1.43±0.25 Hz vs. no coherence: 1.98±0.59 Hz; *P*>0.05), regularity (coherence (mean±SEM): 1.22±0.25 COV-IS vs. no coherence: 1.62±0.54 COV-IS; *P*>0.05), or bursting (coherence (mean±SEM): 24.8±11.6% vs. no coherence: 27.4±7.6%; *P*>0.05).

### Correlation of rhythmic cortical activity with firing of non-5-HT DRN neurons

**Fig. 6:**
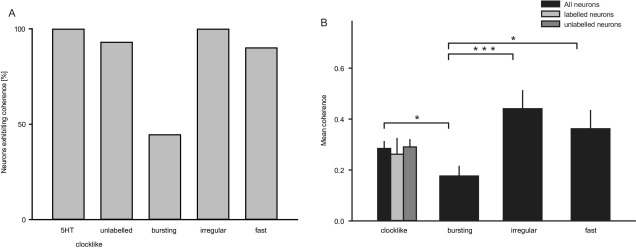
Coherence between firing and cortical oscillation in the different DRN neuronal groups. (A) Percentage of neurons exhibiting significant coherence between the single-unit activity and the cortical slow oscillation in the predominant frequency domains of the cortical slow oscillations (0.5–1.5 Hz). (B) Mean peak coherence of all recorded DRN neurons indicating that bursting 5-HT neurons have a significantly lower mean peak coherence ∼1 Hz frequency domain than clock-like 5-HT and both non-serotonergic neurons groups (ANOVA, * *P*<0.05, *** *P*<0.001, B).

All irregular firing DRN neurons examined (*n*=8/8, 100%) exhibited significant coherence between their firing activity and slow cortical oscillations, with firing being highest during the active component (Fig. 4D–G). Coherence values for this group of neurons were the highest of the four DRN neuron groups examined, indicating a strong relationship with cortical activity (Fig. 6A, B).

The vast majority of fast-firing DRN neurons (*n*=9/10, 90%) exhibited significant coherence between their firing activity and slow cortical oscillations (Fig. 5D–G). Similar to the irregular firing neurons, the activity of the fast-firing neurons was highest during the active component of the slow cortical oscillations (Fig. 5G).

A comparison between the mean peak coherence (in the 0.5–1.5 Hz frequency domain) of the groups revealed a significantly greater coherence in the clock-like serotonergic neurons (and unlabelled clock-like neurons) compared to the bursting serotonergic neurons (Fig. 6B; ANOVA, *P*<0.05). Furthermore the mean peak coherence of the bursting 5-HT neuron group was significantly lower than all the other three groups (*P*<0.05). There was no significant difference between the irregular firing and the fast-firing putative GABA neurons (Fig. 6B; *P*>0.05).

## Discussion

Under deep urethane anaesthesia, the activity of cortical neurons is synchronised and produces stable oscillations, which are qualitatively similar to the activity observed during deep stages of slow-wave sleep in mammals (Steriade, 2006). SWA is expressed by large amplitude (>400 μV) cortical slow oscillation (∼1 Hz), as well as ∂ (1–4 Hz) and spindle (7–14 Hz) oscillations (Steriade, 2000). The ‘active component’ of the slow wave oscillation is expressed by a highly synchronous spike discharges in cortical neurons resulting in a long peak with spindle activity. The trough in the slow oscillations during which high-frequency oscillations are weakest, or absent, will be referred to as the ‘inactive component’. This terminology is used in preference to ‘up’ and ‘down’ states as these generally refer to membrane states of individual neurons as recorded intracellularly (Mallet et al., 2008; Mena-Segovia et al., 2008).

Our findings indicate that DRN neurons show firing activity that is related to cortical slow oscillations depending on their electrophysiological and neurochemical characteristics. Not only is the activity of identified 5-HT neurons linked to the inactive phase of the cortical slow oscillations, but also that non-5-HT neurons are linked to the alternate active phase. This result suggests a divergence in the anatomical innervation of these subsets of dorsal raphe neurons.

We identified four different subgroups of neurons within the DRN and correlated their firing activity with slow cortical oscillations: neurons with slow, regular firing properties (clock-like; including identified 5-HT neurons), 5-HT neurons firing rapid stereotypical bursts (bursting 5-HT neurons), and two groups of non-serotonergic neurons, one with irregular firing rates indicated by a high COV-IS and one with fast-firing neurons (putatively GABAergic). This is in agreement with previous reports (Allers and Sharp, 2003; Kocsis et al., 2006; Hajós et al., 2007; Schweimer and Ungless, 2010).

The diversity of these subgroups was not only expressed by their distinct firing patterns but also by their divergent correlation to cortical activity. Our results indicate that the firing of the majority of neurons within the DRN is related to cortical slow oscillations. Cortical slow oscillations are generated by synchronous, rhythmic depolarising (active component) and hyperpolarising (inactive component) transitions in the membrane potential of principal cortical neurons (Steriade, 2000). This leads to synchronous discharges in corticofugal pathways, which entrain subcortical structures like the basal ganglia, the pedunculopontine nucleus, the locus coeruleus, and the thalamus and most likely also the DRN (Magill et al., 2000, 2001; Steriade, 2000; Mena-Segovia et al., 2008; Eschenko et al., in press). Cortical slow oscillations under urethane anaesthesia are similar to those seen during natural sleep (Steriade, 2006). Nonetheless, an important future challenge will be to conduct similar experiments in unanaesthetised animals during different brain states.

Here we show that distinct DRN neuronal groups exhibit distinct firing patterns in relation to the cortical slow oscillations. The majority of the serotonergic DRN neurons, including identified clock-like and a subset of bursting 5-HT neurons, exhibited significant coherence with the cortical oscillations, and they generally had a higher discharge rate during the inactive component of the slow oscillations. Interestingly, half of the bursting 5-HT neurons did not exhibit significant coherence, which implies that these neurons might not be innervated by the neocortex and firing independently of slow oscillation network.

In contrast to the 5-HT neurons, non-5-HT neurons fire at a higher rate during the active components of the slow waves. This inversely related spike timing between non-5-HT and 5-HT neurons could be undertaken by local connectivity between both populations and thus reinforcing differences in spike timing. Indeed, there is strong anatomical connection of the prefrontal cortex to the DRN, the strongest input to the DRN comes from ventral parts of the medial prefrontal cortex (Hajós et al., 1998; Gonçalves et al., 2009). More specific, glutamatergic prefrontal afferents influence DRN neuron activity by targeting GABA neurons within the DRN, which form inhibitory synapses with 5-HT neurons (Celada et al., 2001; Varga et al., 2001; Jankowski and Sesack, 2004). In addition, stimulation of the prefrontal cortex inhibits putative clock-like 5-HT neurons, and excites putative GABAergic neurons (Hajós et al., 1998; Varga et al., 2001). Taken together, this suggests that the cortical oscillations might influence the spike timing of DRN neurons via these afferents.

In regards to other network oscillations, previous studies have confirmed a relationship between DRN neuron firing and hippocampal theta rhythm (Kocsis and Vertes, 1992, 1996; Kocsis et al., 2006). Kocsis and Vertes (1996) reported that under urethane anaesthesia, subsets of midbrain raphe fire in synchrony with spontaneous and induced hippocampal theta oscillations, but that these neurons did not display characteristic 5-HT neuron features. More recently, Kocsis et al. (2006) found that the firing of classic clock-like 5-HT neurons is not related to hippocampal theta, but that there are subsets of faster firing 5-HT neurons which exhibit strong theta-rhythmic activity.

Our findings further support the idea that discrete subgroups of DRN 5-HT neurons are functionally heterogeneous (Sakai and Crochet, 2001; Waterhouse et al., 2004; Kocsis et al., 2006; Urbain et al., 2006; Nakamura et al., 2008; Ranade and Mainen, 2009). For example, we previously found that noxious footshocks typically evoked rapid excitations in clock-like 5-HT neurons and phasic inhibitions in bursting 5-HT neurons (Schweimer and Ungless, 2010). These divergent responses may also be reflected in the lower percentage of bursting 5-HT neurons firing in synchrony with the cortical slow oscillations.

In conclusion, here we show that DRN neurons exhibit firing activity that is related to cortical slow wave oscillations in distinct ways depending on their electrophysiological and neurochemical identity. Understanding the complexity of the 5-HT system, neuron diversity in the DRN and its network functions is important for the comprehension of the pathophysiology of the 5-HT system, including mood disorder and schizophrenia.
